# Supplementary material for: Phagocytic Activities of Reactive Microglia and Astrocytes Associated with Prion Diseases Are Dysregulated in Opposite Directions
Source: Cells. 2021 Jul 8;10(7):1728. doi: 10.3390/cells10071728 (PMC8304827; doi:10.3390/cells10071728)
Supplement: Supplementary file 1 [file cells-10-01728-s001.zip › cells-1271328-supplementary.pdf]

Figure S1

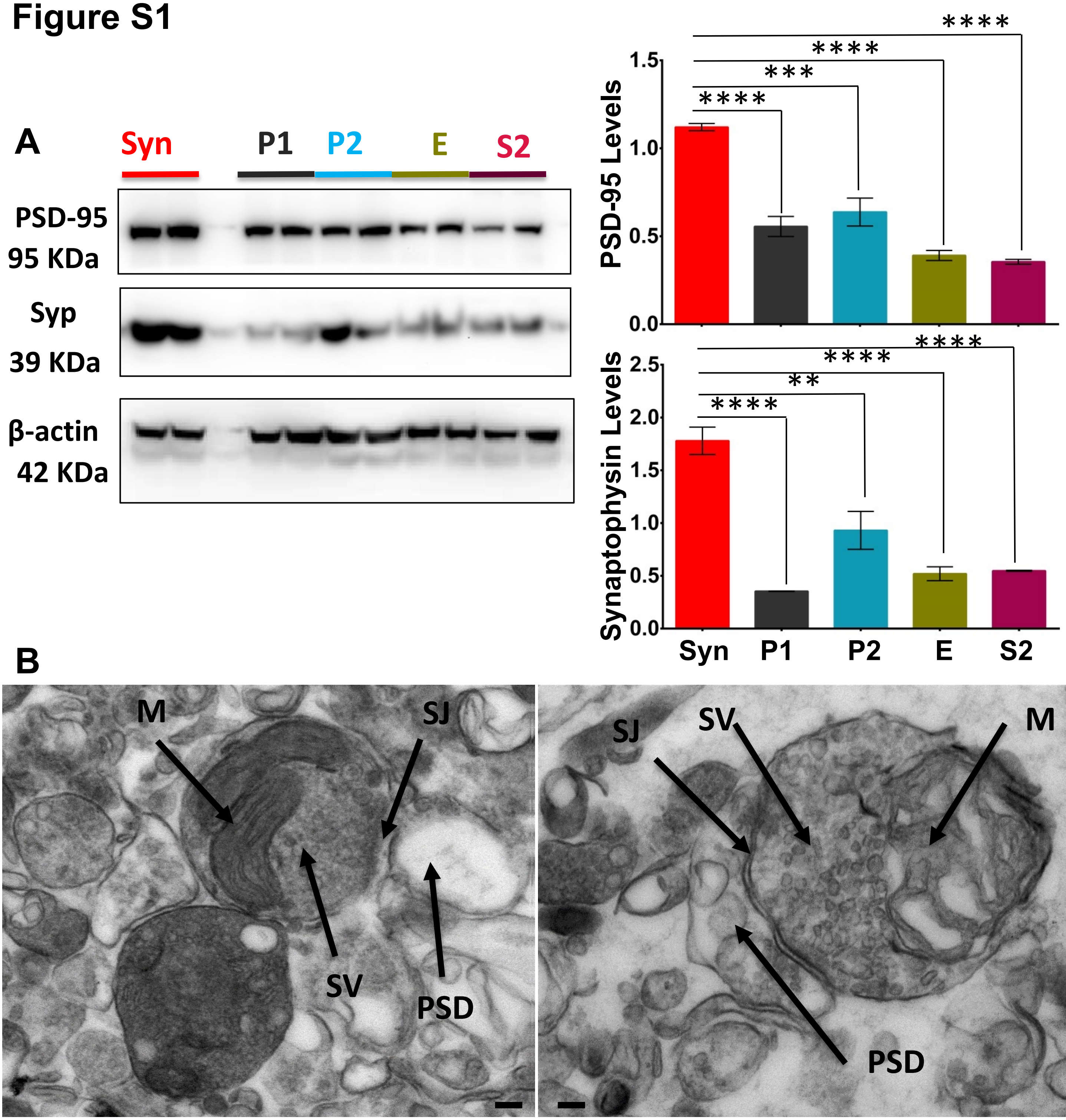

Figure S1. Isolation and characterization of synaptosomes. **A.** Representative Western blots of fractions (P1 - pellet 1, P2 - pellet 2, E - Extrasynaptic fraction and S2 - cytosolic fraction) collected during isolation of synaptosome and the final synaptosome-enriched fraction (Syn) (left), and densitometric analysis of PSD-95 and synaptophysin (Syp) normalized per amounts of β-actin in different fractions. n=3 independent isolations from individual animals, each analyzed in triplicate. Data are expressed as mean ± SEM, n=3 independent synaptosomes preparations isolated from individual control animals, $^{**}p < 0.01$, $^{***}p < 0.001$, $^{****}p < 0.0001$ by one-way ANOVA followed by Tukey's test. **B.** Imaging of synaptosome preparations isolated from control animals by TEM. PSD, postsynaptic density; SJ, synaptic junction; SV, synaptic vesicles and M, mitochondria. Scale bar = 0.1 µm.

Figure S2

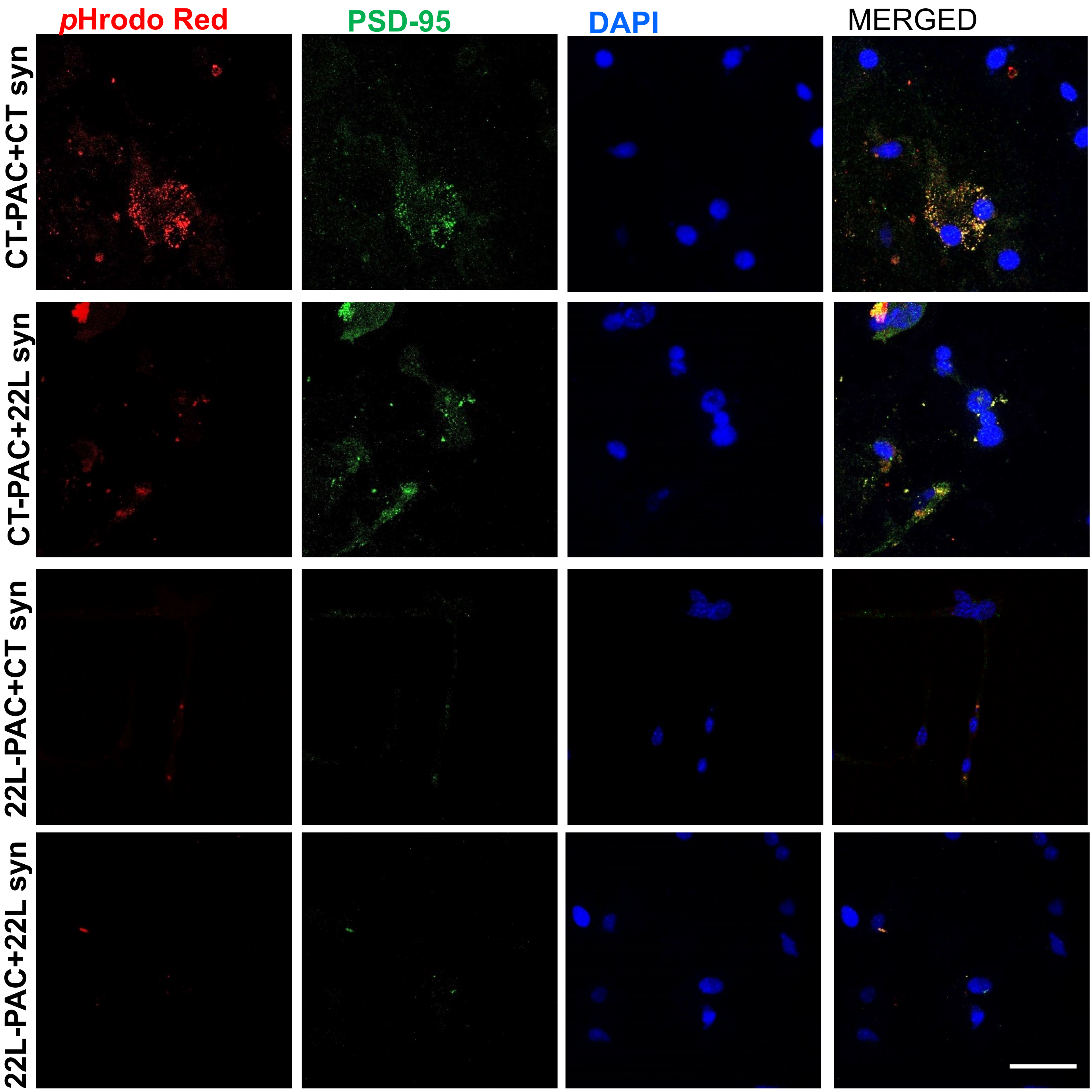

Figure S2: Confocal microcopy imaging of CT-PAC and 22L-PAC incubated with *p*Hrodo Red-conjugated CT-syn or 22L-syn for 24 hours and stained for PSD-95 (green). Cell nuclei are stained with DAPI (blue). Scale bar = 25 μm.
